# Supplementary material for: Rhythm Control and Cardiovascular or Cerebrovascular Outcomes in Patients with Atrial Fibrillation: A Study of the CODE-AF Registry
Source: J Clin Med. 2023 Jul 10;12(14):4579. doi: 10.3390/jcm12144579 (PMC10380641; doi:10.3390/jcm12144579)

SUPPLEMENTARY MATERIAL

Rhythm control and cardiovascular or cerebrovascular outcomes in patients with atrial fibrillation

Ho-Gi Chung; Junbeom Park; Jin-Kyu Park; Ki-Woon Kang; Jaemin Shim; Jin-Bae Kim; Jun Kim; Eue-Keun Choi; Hyung Wook Park; Young Soo Lee; Boyoung Joung

**Contents**	**Page**
**Supplementary Table S1.** Subgroup analysis of primary outcome in rate- and rhythm-control group before overlap weighting.	2
**Supplementary Table S2.** Baseline demographic characteristics between non-RFCA versus RFCA group in patients with rhythm control.	4
**Supplementary Figure S1.** Cumulative incidence of primary outcome in rhythm and rate control treatment groups before overlap weighting.	6
**Supplementary Figure S2.** Cumulative incidence of secondary outcomes in rhythm and rate control treatment groups before overlap weighting.	7

Supplementary Table S1. Subgroup analysis of primary outcome in rate- and rhythm-control group before overlap weighting.

	Rate control (N=4,506)			Rhythm control (N=2,164)					
	Event, n	PYRs	Event/100 PYRs	Event, n	PYRs	Event/100 PYRs	HR (95% CI)	P-value	P for interaction
Sex									
Male (N=4,152)	123	7,184	1.7	52	3,443	1.5	0.82 (0.59~1.14)	0.233	0.252
Women (N=2,518)	88	4,854	1.8	20	1,771	1.1	0.57 (0.35~0.93)	0.023	
Age									
Age<75 (N=4,536)	100	7,803	1.3	48	4,031	1.2	0.86 (0.61~1.22)	0.398	0.570
Age≥75 (N=2,134)	111	4,236	2.6	24	1,183	2.0	0.73 (0.47~1.13)	0.161	
Onset of AF									
<3 month (N=1,263)	17	859	2.0	8	424	1.9	0.93 (0.40~2.15)	0.857	0.376
≥3 month (N=5,407)	194	11,179	1.7	64	4,790	1.3	0.71 (0.54~0.94)	0.019	
CHA_2DS_2-VASc score									
≤2 (N=2,742)	45	4,575	1.0	23	2,760	0.8	0.74 (0.45~1.23)	0.242	0.598
≥3 (N=3,928)	166	7,464	2.2	49	2,454	2.0	0.87 (0.63~1.19)	0.376	
Previous stroke history									
Yes (N=1,231)	69	2,228	3.1	19	825	2.3	0.75 (0.45~1.25)	0.275	0.967
No (N=5,432)	142	9,799	1.5	53	4,384	1.2	0.76 (0.55~1.04)	0.084	
HF history									
Yes (N=791)	39	1,306	3.0	16	613	2.6	0.81 (0.45~1.46)	0.491	

No (N=5,836)	170	10,646	1.6	55	4,578	1.2	0.70 (0.52~0.95)	0.021	0.357
Left atrial diameter (LAD)									
LAD≤4cm (N=1,551)	41	2,445	1.7	17	1,360	1.3	0.75 (0.43~1.33)	0.327	0.278
LAD>4cm (N=3,993)	144	6,925	2.1	44	2,771	1.6	0.76 (0.54~1.06)	0.104	
LAVI (mL/m^2)									
LAVI≤36 (N=1,202)	34	2,096	1.6	10	908	1.1	0.67 (0.33~1.36)	0.268	0.934
LAVI>36 (N=2,942)	118	5,182	2.3	30	1,944	1.5	0.66 (0.45~0.99)	0.045	
LVEF									
LVEF≤40 (N=310)	12	474	2.5	3	250	1.2	0.48 (0.14~1.71)	0.258	0.490
LVEF>40 (N=5,329)	175	9,058	1.9	59	3,967	1.5	0.77 (0.57~1.03)	0.081	

AF = atrial fibrillation; CI = confidence interval; HF = heart failure; HR = hazard ratio; LAVI = left atrial volume index; LVEF = left ventricular ejection fraction; PYRs = person-years.

Supplementary Table S2. Baseline demographic characteristics between non-RFCA versus RFCA group in patients with rhythm control.

	Non-RFCA (N=1,742)	RFCA (N=422)	P-value
Age in years	66.5 (12.6)	64.1 (9.8)	0.001
Male	1,129 (64.8)	310 (73.6)	0.002
Body mass index, kg/m2	24.8 (3.2)	25.3 (3.2)	0.017
Systolic BP, mmHg	124.8 (15.8)	124.1 (13.5)	0.606
Diastolic BP, mmHg	75.4 (12.0)	74.9 (11.0)	0.706
Heart rate, beats/min	76.3 (17.4)	72.5 (15.4)	0.001
Type of AF			0.041
Paroxysmal	1,081 (62.1)	237 (56.3)	
Persistent	639 (36.7)	183 (43.5)	
Permanent	22 (1.3)	1 (0.2)	
Onset of AF			0.001
<3 month	409 (23.5)	24 (5.7)	
≥3 month	1,333 (76.5)	397 (94.3)	
Alcohol intake*	518 (29.7)	128 (30.4)	0.974
Current smoking	580 (33.3)	136 (32.3)	0.720
CHA_2DS_2-VASc score	2.6 (1.6)	2.3 (1.5)	0.001
HAS BLED Score**	1.8 (1.1)	1.5 (1.0)	0.001
Hypertension	1,138 (65.4)	274 (65.1)	0.648
Diabetes	467 (26.8)	103 (24.4)	0.783
Dyslipidemia	587 (33.7)	127 (30.2)	0.329
Myocardial infarction	67 (3.9)	10 (2.4)	0.659
Congestive heart failure	214 (12.3)	44 (10.5)	0.566
Peripheral vascular disease	102 (5.9)	26 (6.2)	0.985
Stroke	270 (15.5)	64 (15.2)	0.201
CKD***	157 (9.0)	34 (8.1)	0.983
Medications			

NOAC	1,308 (75.1)	321 (76.2)	0.751
Warfarin	518 (29.7)	125 (29.5)	0.805
Antiplatelet	261 (15.0)	46 (10.9)	0.092
Beta-blocker	802 (46.1)	225 (53.4)	0.016
CCB	458 (26.3)	94 (22.3)	0.203
Digitalis	60 (3.4)	5 (1.2)	0.050
Diuretics	127 (7.3)	30 (7.1)	0.953
ACEi/ARB	735 (42.2)	155 (36.8)	0.063
Statin	642 (36.9)	161 (38.2)	0.377

Values are presented as mean (standard deviation) or number (%).

*Social drinking and drinking were considered as alcohol intake.

**Modified HAS-BLED=hypertension, 1 point: >65 years old, 1 point: stroke history, 1 point: bleeding history or predisposition, 1 point: liable international normalized ratio, not assessed: ethanol or drug abuse, 1 point: drug predisposing to bleeding, 1 point.

***CKD was defined as eGFR<60mL/min/1.73m^2

ACEi = angiotensin converting enzyme inhibitor; AF = atrial fibrillation; ARB = angiotensin II receptor blocker; BP = blood pressure; CCB = calcium channel blocker; CKD = chronic kidney disease; NOAC = non-vitamin K antagonist oral anticoagulant.

Supplementary Figure S1. Cumulative incidence of primary outcome in rhythm and rate control treatment groups before overlap weighting. Primary outcome was a composite of death from cardiovascular causes, stroke, or hospitalization with worsening of heart failure or acute coronary syndrome.

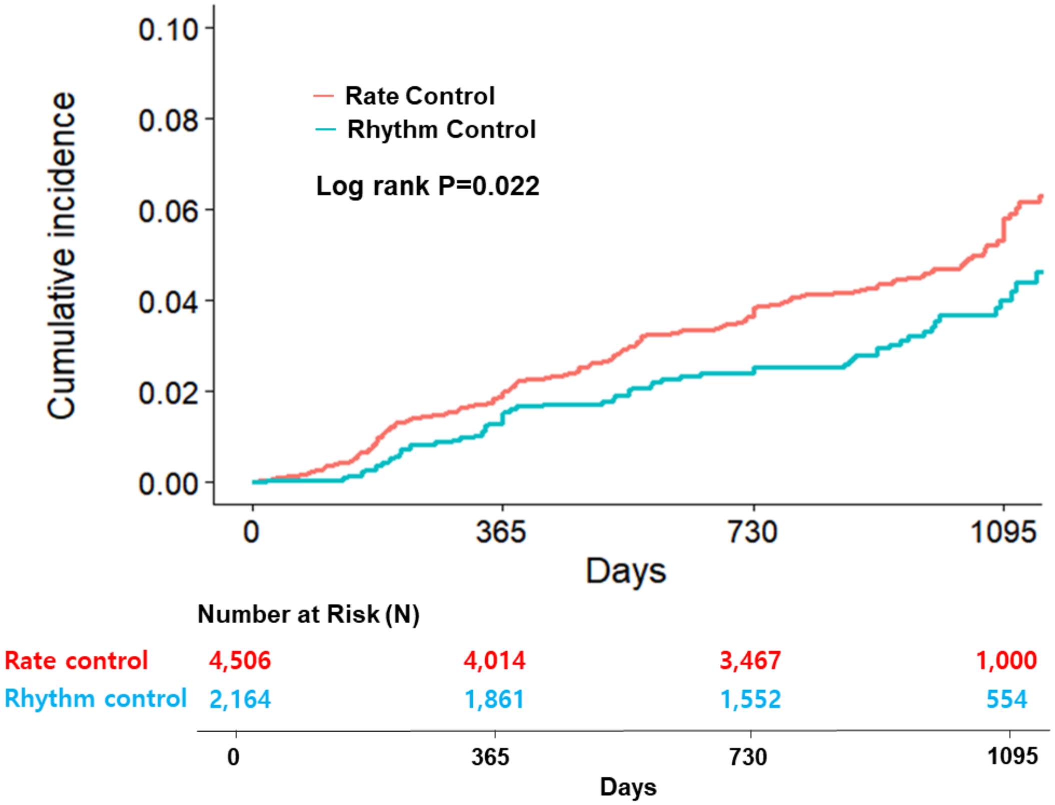

Supplementary Figure S2. Cumulative incidence of secondary outcomes in rhythm and rate control treatment groups before overlap weighting. Death from cardiovascular cause (A), Stroke (B), Hospitalization with worsening of heart failure (C), and Acute coronary syndrome (D).

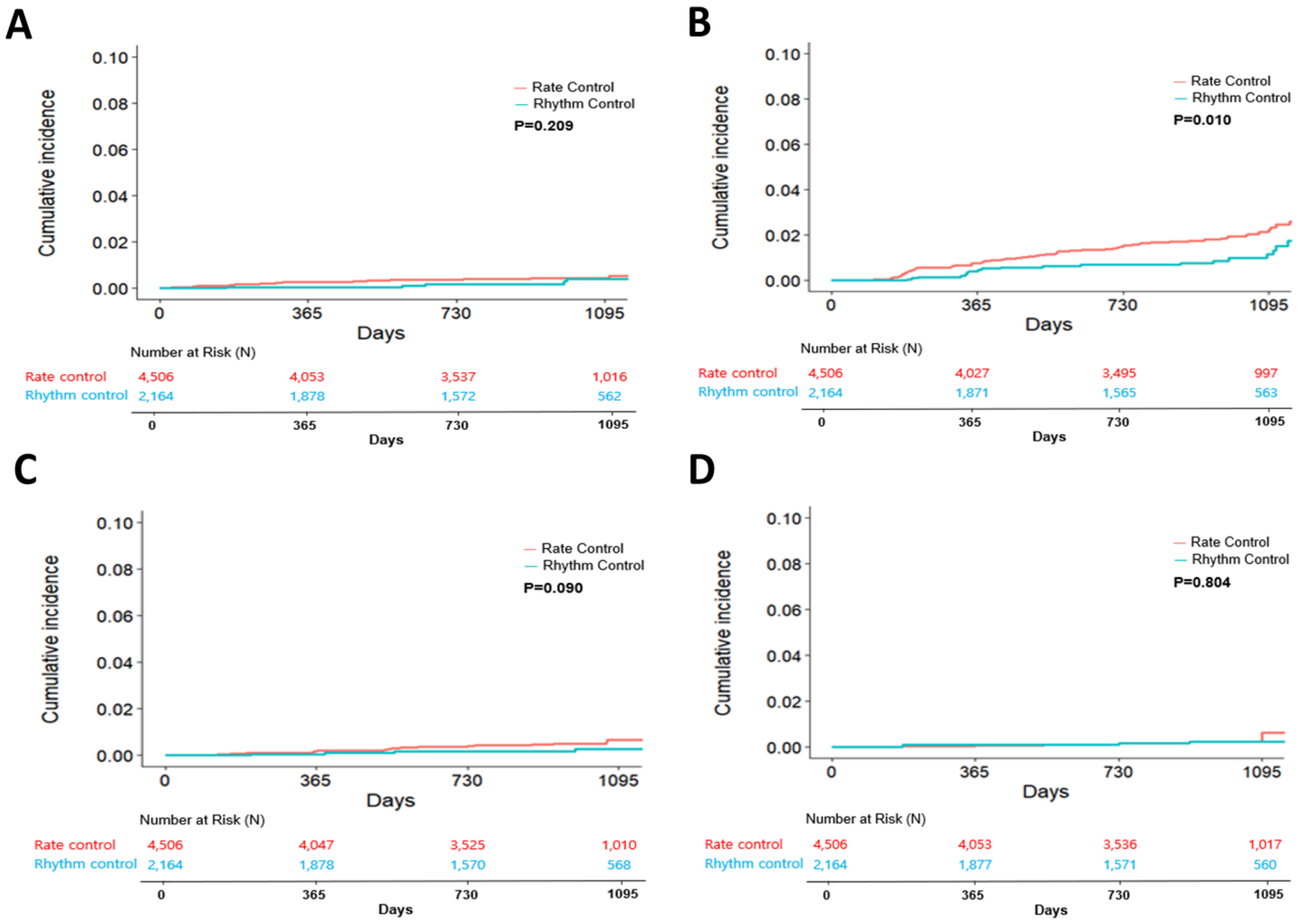

Supplement: Supplementary file 1 [file jcm-12-04579-s001.zip › jcm-2469838-supplementary.pdf]
